# High Pressure Synthesis of p-Type Ce_*y*_Fe_4−*x*_Co_*x*_Sb_12_ Skutterudites

**DOI:** 10.3390/ma9040257

**Published:** 2016-03-31

**Authors:** Yadi Liu, Xiaohui Li, Qian Zhang, Long Zhang, Dongli Yu, Bo Xu, Yongjun Tian

**Affiliations:** State Key Laboratory of Metastable Materials Science and Technology, Yanshan University, Qinhuangdao 066004, Hebei, China; ready1986@163.com (Y.L.); lixiaohui.hebei@163.com (X.L.); qian.zhang@hpstar.ac.cn (Q.Z.); lzhang@ysu.edu.cn (L.Z.); ydl@ysu.edu.cn (D.Y.); fhcl@ysu.edu.cn (Y.T.)

**Keywords:** skutterudites, high pressure synthesis, thermoelectric properties, filling fraction, substitution

## Abstract

Co-substituted p-Type CeFe_4−*x*_Co_*x*_Sb_12_ skutterudites were successfully synthesized with a high pressure synthesis method. The structure, composition, and thermoelectric properties were investigated. The obtained Ce_*y*_Fe_4−*x*_Co_*x*_Sb_12_ samples show the skutterudite structure of $Im\bar{3}$ symmetry. The hole concentration decreases with elevating Co substitution level, leading to increased Seebeck coefficient and electrical resistivity. Meanwhile, the filling fraction of Ce decreases, which is unfavorable for reducing the lattice thermal conductivity. As a result, the thermoelectric performance of Ce_*y*_Fe_4−*x*_Co_*x*_Sb_12_ deteriorates with higher Co content. The maximal *ZT* of 0.91 was achieved at 763 K for the optimal Ce_0.92_Fe_4_Sb_12_ sample.

## 1. Introduction

Thermoelectric materials have attracted great research interest during the past few decades due to their prospective applications in energy conversion to alleviate the greenhouse effect and environmental pollution [1]. In general, the performance of thermoelectric materials is characterized by the dimensionless figure of merit *ZT*

(1) $$ZT=S^{2}T/\rho \kappa$$

where *S*, *T*, *ρ*, and κ are the Seebeck coefficient, temperature, electrical resistivity, and thermal conductivity, respectively. To make the thermoelectric device competitive with other energy conversion devices, thermoelectric materials with high *ZT* in the working temperature range must be fabricated, which is a practical challenge because of the entanglements between *S*, *ρ*, and κ. Improvement of one parameter usually adversely influences others. To unravel the entanglements, Slack proposed the concept of phonon-glass electron-crystal (PGEC) in 1995 [2]. Skutterudites, a prototype of the PGEC concept, are among the most promising thermoelectric materials working in the intermediate temperature region [3].

Various approaches have been taken to improve the thermoelectric properties of skutterudites, such as void filling [4,5,6,7,8,9,10,11,12] and lattice atom substituting [13,14,15,16,17,18,19]. Compared with n-type CoSb_3_-based skutterudites for which *ZT* records are constantly refreshed [4,5,6], the developments in p-type skutterudites relatively lag behind [9,10]. Elemental filled FeSb_3_-based skutterudites are one of the most important p-type skutterudites. In this class of materials, κ is greatly suppressed due to the rattling filler atoms (with a near unit filling fraction), and a low *ρ* is ensured by the high hole concentration (>10^21^ cm^−3^); meanwhile, a moderate *S* is maintained due to the large effective mass of hole [20]. Further enhancement of *ZT* for elemental filled FeSb_3_-based skutterudites might be possible through enhancing power factor *PF* and suppressing thermal conductivity.

(2) $$PF=S^{2}/\rho$$

Substituting framework atoms with electron-richer atoms (such as Co/Ni for Fe) would be a possible method. By such kind of substitutions, the hole concentration can be lowered to benefit enhancement of *S*. In addition, the impurity scattering of phonons due to mass fluctuation may contribute to further lower κ.

The effects of Co/Ni substitution for Fe on the thermoelectric properties of CeFe_4_Sb_12_ were investigated previously [21,22,23,24]. In these studies, the time-consuming solid state reaction method was employed to fabricate the samples, which usually takes several days. Recent works have highlighted pressure as a fundamental thermodynamic variable in synthesizing elemental filled CoSb_3_ [25,26,27] and Te-doped CoSb_3_ [28,29,30]. High pressure can lower the reaction temperature and facilitate the synthesis of metastable phase by shifting reaction equilibrium [31]. In this work, Co-substituted CeFe_4_Sb_12_ skutterudites were synthesized by using a time-saving high pressure synthesis (HPS) method. We found that the actual filling fraction of Ce decreases slightly with elevating Co substitution level, and a notable decrease in the hole concentration is observed. The hole concentration decrease shows a more pronounced effect on the electrical resistivity, leading to a significantly reduced power factor in the sample with the highest Co level. As a result, the thermoelectric performance deteriorates with higher Co content. The optimal Ce_0.92_Fe_4_Sb_12_ showed a maximal *ZT* of 0.91 at 763 K.

## 2. Experimental Methods

Ce (99.9%), Fe (99.5%), Co (99.8%), and Sb (99.999%) powders were mixed according to the atomic ratio of 1:(4−*a*):*a*:12 (*a* = 0, 0.5, and 1, respectively). The mixture was filled into a mold and shaped into a cylinder with a cold press method. The as-prepared cylinder was inserted into an *h*-BN crucible and loaded into a high pressure apparatus for HPS experiments. The first step of HPS was carried out at 3 GPa and 1100 K for 0.5 h. The intermediate product was ground into powders under argon protection, shaped and subject to the second step of HPS (3 GPa and 873 K for 3 h). The obtained product was ground into powders, washed in acidic solution to remove excess Ce, and sintered into dense pellets by using spark plasma sintering (SPS) at 60 MPa and 853 K for 15 min. Rectangular blocks (2 × 2 × 8 mm^3^), disks (ø 6 mm × 1 mm), and slices (2.5 × 6 × 0.4 mm^3^) were cut from the final pellets for the electrical transport, thermal conductivity, and Hall coefficient measurements, respectively.

The chemical composition and crystal structure of the final products were characterized with electron probe microanalysis (EPMA, JEOL JXA-8230, JEOL, Tokyo, Japan) and X-ray diffraction (XRD, Rigaku D/MAX/2500/PC, Rigaku, Tokyo, Japan), respectively. The Seebeck coefficient and electrical resistivity were measured with a ZEM-3 apparatus (Ulvac-Riko, Yokohama, Japan) and the thermal conductivity was measured with a TC-9000H apparatus (Ulvac-Riko, Yokohama, Japan). The uncertainty in *S*, ρ, and κ measurements is within 5%. The Hall coefficient measurements were performed at room temperature with a Physical Property Measurement System (PPMS, Quantum Design, San Diego, CA, USA). The heat capacity measurements were carried out between 5 and 25 K with the heat capacity option of PPMS.

## 3. Results and Discussion

**Figure 1:**
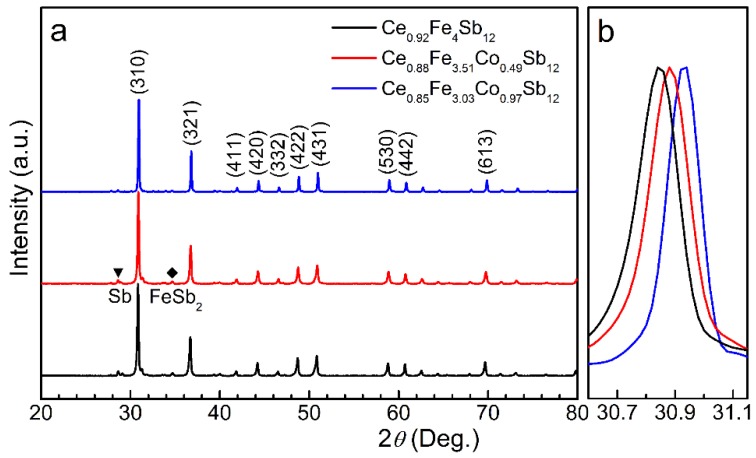
(**a**) XRD patterns (Cu K_α_) for Ce_*y*_Fe_4−*x*_Co_*x*_Sb_12_ samples; (**b**) the magnified (013) peaks emphasizing the continuous lattice parameter contraction with elevating Co substitution level.

XRD patterns from Ce_*y*_Fe_4−*x*_Co_*x*_Sb_12_ samples are shown in Figure 1. All these samples are dominated with a skutterudite structure of $Im\bar{3}$ symmetry. XRD results also reveal trace amounts of FeSb_2_ and Sb, which are less than 5% as determined by EPMA analyses. A systematical shift of diffraction peaks to higher diffraction angles occurs with increasing Co content, as illustrated in Figure 1b for the magnified (013) peak, indicating that the lattice parameter decreases with elevating Co level. The actual content of Co (*x*) and filling fraction of Ce (*y*) in Ce_*y*_Fe_4−*x*_Co_*x*_Sb_12_ samples determined by EPMA measurements are listed in Table 1. The highest filling fraction of Ce of 0.92 is reached in the sample without Co substitution (Ce_0.92_Fe_4_Sb_12_), which is at the same level as that of 0.91 achieved in an ambient-pressure synthesized sample [11]. Cobalt atom has one more valence electron and smaller atomic size than those of iron atoms. By substituting Co on Fe sites, extra electrons from Co are introduced to the framework meanwhile the lattice parameter is reduced. Both effects contribute adversely to the void-filling process of Ce. Therefore, the higher Co substitution level, and the lower Ce filling fraction [24], and both contribute to a smaller lattice parameter. The actual filling fraction of Ce in our HPS samples decreases slightly with increasing Co substitution level, and is higher than that in the ambient-pressure synthesized sample with the same Co level [24], demonstrating an advantage of high pressure for achieving higher filling fraction.

Room temperature electrical transport properties for different samples are summarized in Table 1. All samples exhibit positive Seebeck coefficient, indicating the majority charge carriers of holes. The hole concentration deduced from the measured Hall coefficient decreases notably with elevating Co substitution level, which is due to Co substitution introducing extra electrons into the system. In addition, the hole concentration in our Ce_0.85_Fe_3.03_Co_0.97_Sb_12_ sample is 9.5 × 10^20^ cm^−3^, less than half of that in the ambient-pressure synthesized Ce_0.9_Fe_3_CoSb_12_ (nominal composition) [21]. The difference can be attributed to the higher filling fraction of Ce in our HPS sample, and accounts for the significant increase in the electrical resistivity of our HPS Ce_0.85_Fe_3.03_Co_0.97_Sb_12_ sample. The hole concentrations can also be calculated by simple charge counting based on the chemical compositions, which differ from those listed in Table 1. A similar difference was previously reported [11,32], and might be related to the multiple bands near the Fermi level in elemental-filled FeSb_3_ materials where a simple relationship between carrier concentration and Hall coefficient does not exist. Note that the carrier mobility is continually suppressed with increasing Co level, indicating a decreased hole mean free path caused by impurity scattering.

**Figure 2:**
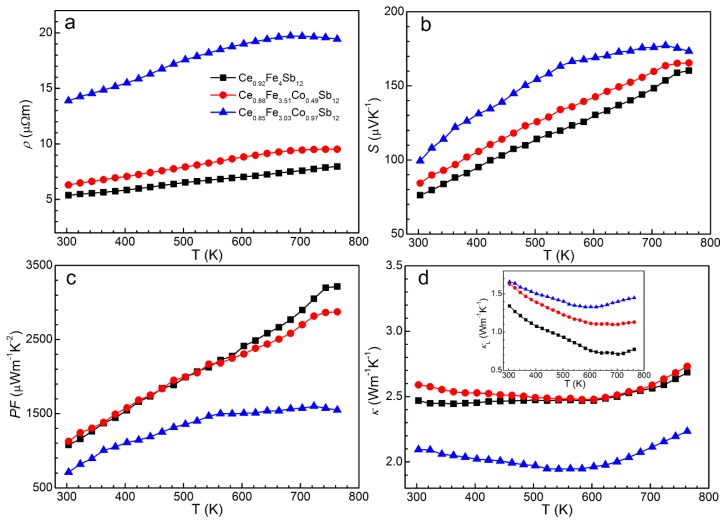
Temperature dependent thermoelectric properties of Ce_*y*_Fe_4−*x*_Co_*x*_Sb_12_ samples. (**a**) electrical resistivity *ρ*; (**b**) the Seebeck coefficient *S*; (**c**) power factor *PF*; and (**d**) thermal conductivity κ. The inset to panel d shows the lattice thermal conductivity κ_L_.

The temperature dependent thermoelectric properties of Ce_*y*_Fe_4−*x*_Co_*x*_Sb_12_ samples are shown in Figure 2. The electrical resistivity increases with elevating Co substitution level (Figure 2a), which can be attributed to the decreased hole concentration compensated by the additional electrons from cobalt. The most Co-substituted Ce_0.85_Fe_3.03_Co_0.97_Sb_12_ exhibits the largest electrical resistivity, which starts to decrease at high temperature due to thermally activated carriers. The other two samples with lower Co substitution level show a heavily doped semiconductor behavior and the resistivities increase with elevating temperature due to stronger scattering. As the hole concentration decreases with higher Co substitution level, an increase in the Seebeck coefficient occurs (Figure 2b). While the most Co-substituted Ce_0.85_Fe_3.03_Co_0.97_Sb_12_ shows an early onset of intrinsic regime of conduction occurring at *ca.* 700 K, the Seebeck coefficient for the other two samples increases monotonically with elevating temperature. The tendencies of the Seebeck coefficient with respect to temperature and Co substitution level are consistent with those of the electrical resistivity.

The temperature dependent power factor is presented in Figure 2c. Compared with the increase of *S* as a function of Co substitution level, the increase of *ρ* is more significant, leading to decreased peak values of *PF* with increasing Co substitution level. The maximal *PF* of 3200 µW m^−1^ K^−2^ is achieved for Ce_0.92_Fe_4_Sb_12_, which is comparable to the best values of other single elemental filled FeSb_3_ skutterudites [11]. We note a remarkably reduced *PF* in Ce_0.85_Fe_3.03_Co_0.97_Sb_12_ sample. In this sample, the actual filling fraction of Ce is higher than that in the ambient-pressure synthesized sample with the same Co level [24], leading to a lower hole concentration and a significantly increased electrical resistivity.

The temperature dependence of thermal conductivity is shown in Figure 2d for Ce_*y*_Fe_4−*x*_Co_*x*_Sb_12_ samples. The variation tendency of κ with respect to temperature is similar to that of Ce_*y*_Fe_4-(x/2)_Ni_x/2_Sb_12_ samples [24]. The total thermal conductivity is a sum of the lattice thermal conductivity (*κ*_L_) and the carrier contribution κ_c_,

(3) κ = κ_L_ + κ_c_

κ_c_ can be evaluated with the Wiedemann−Franz law,

(4) κ_c_ = *LT*/ρ

where *L* is the Lorenz number and a value of 2 × 10^−8^ WΩ K^−2^ is used here [33]. The temperature dependent κ_L_ can then be estimated and is shown as the inset of Figure 2d. The minimum κ_L_ of 0.71 Wm^−1^ K^−1^ is reached in Ce_0.92_Fe_4_Sb_12_ at 700 K. For all the samples, κ_L_ first decreases with increasing temperature, and then increases slightly at high temperature due to the bipolar effect. The onset of bipolar effect shifts to higher temperature for the sample with lower Co substitution level, which can be attributed to the higher hole concentration suppressing the bipolar effect. For Ce_*y*_Fe_4−*x*_Co_*x*_Sb_12_ samples, it is plausible that both impurity atoms (Co) and rattling atoms (Ce) contribute to phonon scattering and reduce κ_L_. However, κ_L_ increases with elevating Co substitution level. Similar results were previously observed in Ni-doped CeFe_4_Sb_12_ systems [24]. These results indicate that impurity scattering of phonon due to mass/force constant fluctuation of Fe and Co is less significant for our samples, and the reduction of κ_L_ is dominated by the rattling fillers: higher filling fraction leads to lower κ_L_.

**Figure 3:**
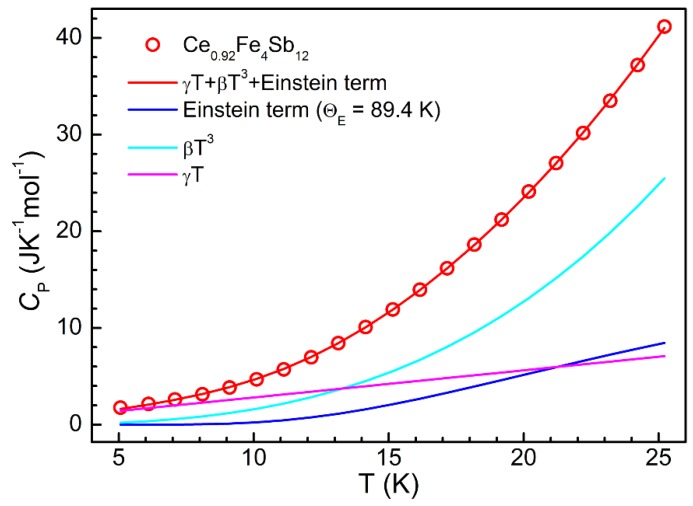
Low-temperature molar heat capacity of Ce_0.92_Fe_4_Sb_12_ (see main text for the fitting details).

One of the effective techniques to confirm such kinds of localized vibrational modes is the low temperature heat capacity (*C*_P_) measurement [34,35,36], which was performed on Ce_0.92_Fe_4_Sb_12_ in the temperature range of 5−25 K. The heat capacity as a function of temperature is well described by the sum of an electronic term, γ*T*, and phonon terms, which are the Einstein term for the loosely bonded filler atom and the Debye *T*^3^ term for the 16 atoms of the framework. The extra Debye *T*^5^ term can be neglected at low temperature [32,37]. As shown in Figure 3, *C*_P_ is well fitted by

(5) $$C_{P}=\gamma T+\beta T^{3}+Ax^{2}e^{x}/{\left(e^{x}-1\right)}^{2},$$

where γ = 0.281 J K^−2^ mol^−1^, β = 0.00159 J K^−4^ mol^−1^, (corresponding to a Debye temperature of 269 K), *A* = 22.05 J K^−1^ mol^−1^, and *x* = Θ_E_/*T* with an Einstein temperature Θ_E_ = 89.4 K. The fitting quality is significantly good with χ^2^ = 5.62 × 10^−3^ and *R*^2^ = 0.99996. The Einstein temperature determined in this work is comparable with that (86 K) reported previously [38]. In addition, the filling fraction determined from *A/3R* (*R* is the ideal gas constant) is 0.88, which is very close to the value of 0.92 from EPMA measurement and indicates successful filling of Ce atoms into FeSb_3_ voids.

**Figure 4:**
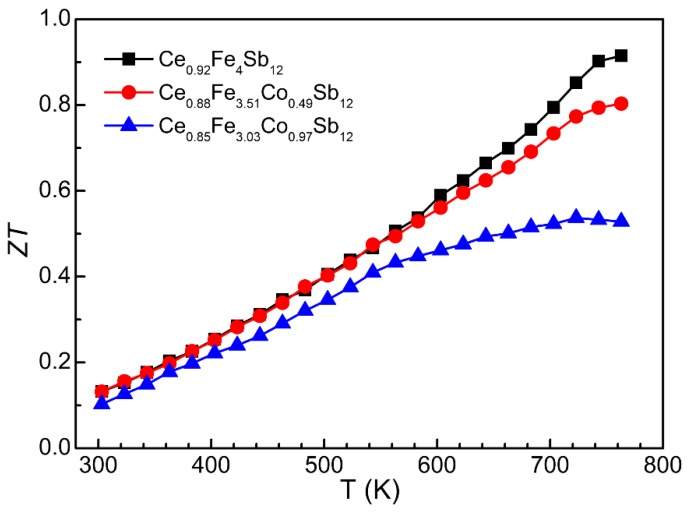
*ZT* as a function of temperature for Ce_*y*_Fe_4−*x*_Co_*x*_Sb_12_ samples.

Figure 4 shows *ZT* of Ce_*y*_Fe_4−*x*_Co_*x*_Sb_12_ samples calculated from the measured thermoelectric properties. For the optimal Ce_0.92_Fe_4_Sb_12_ sample, *ZT* increases with temperature and reaches the highest value of 0.91 at 763 K, which is slightly higher than that achieved in ambient-pressure synthesized sample [11]. It is noted the thermoelectric performance of our HPS Ce_*y*_Fe_4−*x*_Co_*x*_Sb_12_ samples deteriorates with increasing Co substitution level, which differs from those ambient-pressure synthesized samples. For example, the highest *ZT* of 0.9 was achieved in Ce_0.9_Fe_3_CoSb_12_ [21] and CeFe_3.8_Co_0.2_Sb_12_ [22]. In our samples, the actual filling fraction of Ce only slightly decreases with increasing Co content, which leads to a much smaller hole concentration and an undesirable increase in *ρ* compared with those ambient-pressure synthesized samples. Consequentially, an enhancement in thermoelectric performance does not occur through substituting framework atoms with electron-richer atoms alone in our HPS samples.

Nonetheless, HPS methods employed in this study demonstrates an advantage over the solid state reaction method at ambient pressure. HPS significantly accelerates the fabrication process and shortens the total reaction duration to several hours, and allows us to tune the filling fraction in a wider range. In addition, the synthetic pressure and temperature conditions can easily be realized in industrial production [39], another advantage for massive production for thermoelectric applications. Combined with the strategies of multiple filling/substituting and nanostructuring (through, e.g., ball milling and severe plastic deformation) [9,10,40,41], further enhancement of *ZT* values of p-type skutterudites could be realized with the HPS method.

## 4. Conclusions

p-type Ce_*y*_Fe_4−*x*_Co_*x*_Sb_12_ skutterudites were successfully synthesized by using an efficient HPS method within several hours. The highest filling fraction of Ce (*y* = 0.92) was reached in the Co-free sample. With increasing Co substitution level, both hole concentration and Ce filling fraction decrease. The enhancement in *S* due to a smaller hole concentration is offset by the increase in ρ. Moreover, the suppression of κ_L_ seems dominated by the rattling filler atoms, and lower filling fraction of Ce with higher Co content is unfavorable to reduce κ_L_. The maximal *ZT* of 0.91 was achieved in the optimal Ce_0.92_Fe_4_Sb_12_ sample, and the thermoelectric performance of Ce_*y*_Fe_4−*x*_Co_*x*_Sb_12_ deteriorates with higher Co content.

## Figures and Tables

**Table 1:** Co content (*x*), Ce filling fraction (*y*), and room temperature lattice constant (*a*), Seebeck coefficient (*S*), electrical resistivity (*ρ*), carrier concentration (*p*), and carrier mobility (*μ*) for HPS Ce_*y*_Fe_4−*x*_Co_*x*_Sb_12_ samples.

*x*	*y*	*a* (nm)	*S* (μV K^−1^)	ρ (μΩ m)	*p* (10^20^ cm^−3^)	*μ* (cm^2^ V^−1^ s^−1^)
0	0.92 (2)	0.9167 (3)	76.2	5.0	21.5	5.8
0.49 (1)	0.88 (2)	0.9153 (3)	84.3	6.9	17.1	5.3
0.97 (2)	0.85 (2)	0.9140 (3)	99.4	14.3	9.5	4.6
